# Read at home to do well at school: informal reading predicts achievement and motivation in English as a foreign language

**DOI:** 10.3389/fpsyg.2023.1289600

**Published:** 2024-01-23

**Authors:** Jennifer Meyer, Johanna Fleckenstein, Maleika Krüger, Stefan Daniel Keller, Nicolas Hübner

**Affiliations:** ^1^Leibniz Institute for Science and Mathematics Education, Kiel, Germany; ^2^Department of Applied Educational Science, University of Hildesheim, Hildesheim, Germany; ^3^Primary School Pedagogy, Structural Unit Educational Science, University of Potsdam, Potsdam, Germany; ^4^Department Subject Specific Teaching and Learning Science, Zurich University of Teacher Education, Zürich, Switzerland; ^5^Institute of Education, University of Tübingen, Tübingen, Germany

**Keywords:** informal language learning, expectancy-value theory, motivation, English achievement, longitudinal data

## Abstract

**Introduction:**

Learning English as a foreign language is necessary for many students to actively participate in an increasingly globalized world. This study explores the role of informal English language engagement for students’ reading and listening skills, as well as motivation to learn English. In an era of global interconnectedness, informal learning gains importance as a supplement to formal education.

**Methods:**

This study extends the evidence base by analyzing extramural reading and listening activities in a large-scale longitudinal investigation involving secondary school learners (N = 1,994) in Germany.

**Results:**

Our results show that frequent informal reading significantly relates to increases in students’ English comprehension skills and their motivation for language learning, reaffirming previous cross-sectional findings.

**Discussion:**

The results highlight the relevance of informal language activities for effective language learning and students’ English as a foreign language motivation. Additionally, discrepancies between reading and listening outcomes are discussed.

## 1 Introduction

Proficiency in English as a foreign language (EFL) is considered a key factor in educational and vocational success in the modern world. It confers a considerable advantage upon learners, affording economic, social and cultural benefits that are unlike those provided by any other foreign language in the European context (Keller et al., 2020). Accordingly, EFL proficiency is a crucial learning goal of upper secondary education particularly in educational systems where English is not the language of schooling (e.g., Fleckenstein et al., 2016; Hübner et al., 2021). However, learning gains during upper secondary education are small compared to lower secondary education (Hill et al., 2008), which is why it is important to increase understanding of what type of instruction, activities or materials can support student learning during that period.

Existing research has shown that students’ leisure-time activities are related to their academic achievement in different domains, and highlighted associations between formal and informal learning (Eccles et al., 2003; Mol and Bus, 2011; Fredricks, 2012). Such incidental or informal learning typically comprises activities that are unplanned, part of students daily activities and undertaken willingly without a formal requirement (Werquin, 2010; see also Greene et al., 2021). Specifically for EFL learning, previous research has shown positive associations between informal learning during leisure time and student achievement and motivation specifically in English as a foreign language, providing evidence of positive associations (e.g., Kuppens, 2010; Schmitt and Redwood, 2011; Kusyk and Sockett, 2012; Lindgren and Muñoz, 2013; Rodgers, 2013; González-Fernández and Schmitt, 2015; Lai et al., 2015; Sundqvist and Wikström, 2015; Jensen, 2017; Lee and Dressman, 2018; Peters, 2018; Peters and Webb, 2018; Lee, 2019; Lee and Drajati, 2019; Puimège and Peters, 2019; Pujadas and Muñoz, 2019; Krüger, 2023).

In the current study, we provide additional evidence on the role of informal language learning on English as a foreign language (EFL) competencies (grades and standardized tests) in a sample of German students. We do so by employing a research approach that has rarely been used to study this topic: while most extant studies used cross-sectional designs with small sample sizes, we based this study on longitudinal large-scale data with two measurements from observational data in an authentic school environment. This allowed us to control potentially confounding effects of prior achievement, motivation and informal learning activities, providing more precise insights regarding the effects of informal learning (Hübner et al., 2023). Further, our study focuses on learners at upper-secondary level [International Standard Classification of Education (ISCED) Level 3; Organization for Economic Co-operation and Development [OECD], 2015] whereas many previous studies have focused on learners at tertiary level (college and university). Moreover, our dataset enabled us to take specific modalities of language-use into account: we distinguished between informal English reading and listening activities and investigated whether there were differential associations with reading and listening comprehension as measured by standardized tests, conceptualizing formal competencies. Finally, we further substantiate previous evidence on the extent to which engaging with English media can be beneficial for students’ motivational development.

### 1.1 Informal learning in English as a foreign language

Incidental (or informal) learning takes place when students, while pursuing a goal other than learning, encounter material that leads to a cognitive or behavioral change that can result in skill development (Greene et al., 2021). Importantly, incidental or informal learning typically comprises activities that are unplanned, part of students’ daily activities, and undertaken willingly without formal requirements (Werquin, 2010). On the basis of these characteristics, we define informal learning by (1) the nature of the goal that students are following when engaging in an activity (e.g., learning vs. other; see Kelly, 2012) and (2) by the context (formal vs. informal). Informal reading and listening activities are particularly relevant for EFL learning because of the importance of input in foreign language learning in general (Krashen, 1982, 1985, 1989; an overview of studies, see Ellis, 2008, pp. 274–295). In the context of EFL learning, informal learning can be defined as students’ engagement with language input unrelated to schoolwork or homework. The terms “extracurricular” or “extramural” are both used to express this concept in the relevant literature. We use these terms interchangeably in the current study as we aimed to integrate findings from these (seemingly) different research areas.

Within a cognitive-interactionist framework (Long, 2015), both the characteristics of the language input and learners’ individual experiences as they process that input play an important role in triggering the internal processing that leads to construction of new language knowledge. In that context, it is important to distinguish “input” from “intake”: input refers to all the target language that learners read and hear, while intake refers to the part of input which learners comprehend and act upon to develop their internal grammar of the target language, or understanding of its rules of use (Gass, 1997). The rich and authentic language input that learners engage with during informal learning activities can stimulate these processes of intake just as well as the input received during classroom instruction. Books, games and videos in the target language stimulate many of those cognitive processes of engagement that are considered effective for foreign language learning as their neural correlates involve widespread brain regions as a function of rich perceptual environments (Li and Jeong, 2020; Kersten et al., 2023). While the resulting development of language proficiency might be small or large, it will reinforce or change existing schemas, instigating the learner’s active construction of language knowledge and nudging them forward in the process of improving their interlanguage (Selinker, 1972; Kersten et al., 2023). These processes of cognitive-interactional language learning can be seen as the reason why activities such as interacting with others in on-line media, watching the latest YouTube video or gaming with peers in the target language can have a beneficial effect on learning. Just as rich classroom instructions, such activities can lead to the activation of prior knowledge and lead learners to focus on interesting meaningful content. Further, many of these activities might actively involve learners in problem-solving. They therefore provide the kind of multisensory stimuli and real-life contextualization that are crucial to EFL learning both inside and outside of the classroom (Long, 2015; Tedick and Lyster, 2020).

These assumptions have been underscored with empirical evidence (see Supplementary Table 1 in the Supplementary material E for an overview): studies have shown, for example, that when reading or listening to English language content in an informal way more frequently, students might become immersed in the situation, which can be highly effective for second-language acquisition (VanPatten and Williams, 2014). Such learning is similar to the experiences students can have when studying abroad, which has been shown to facilitate students’ language learning (Hübner et al., 2021). More specifically, studies have provided evidence on beneficial effects of informal learning activities, focusing on different aspects of EFL competence: for example, vocabulary learning, speaking, reading and listening competencies. For example, Frumuselu et al. (2015) conducted an experiment with 40 undergraduates, exposing English-as-a-foreign-language students to television series in English; they found positive effects of the intervention on vocabulary learning and comprehension. Vidal (2011) reported on an experiment that showed reading and listening input to influence vocabulary growth. Fievez et al. (2023) investigated effects of watching Netflix with captions in an experimental design showing positive effects for vocabulary learning. Sundqvist and Wikström (2015) found positive associations between digital games and vocabulary learning in a sample of 80 secondary school students. Kuppens (2010) investigated the role that television programs play in the oral competencies of elementary school students (*N* = 374): results indicated positive associations between oral competencies and media activities. Other studies show positive effects of informal learning activities on a range of language competencies in foreign languages for listening, reading, writing, and speaking skills in younger learners and adolescents through reading, surfing the internet, watching films and television series, and gaming (see Supplementary Table 1 in Supplementary material E; e.g., Kuppens, 2010; Verspoor et al., 2011; Vidal, 2011; Sylvén and Sundqvist, 2012; Lindgren and Muñoz, 2013; Frumuselu et al., 2015; Sundqvist and Wikström, 2015; Peters, 2018).

In sum, prior research has provided far-ranging empirical evidence of positive associations between informal learning and engaging with media content, highlighting that different forms of input, output, and interaction with English language content can relate positively to students’ language learning. As illustrated, numerous studies have investigated this topic, however, previous research is limited, as most studies were either based on small samples, focused on university students, and/or were either experimental or did not control for important covariates such as prior achievement or motivation. Additionally, most studies focused on vocabulary, only little research is available considering reading and listening comprehension or school grades. Thus, it remains largely unclear if results found in earlier studies generalize to authentic school setting and to what extent informal learning practices benefit specific EFL-skills such as reading and listening comprehension as well as subject-specific motivation and school grades.

### 1.2 The role of modality in informal EFL learning: focus on reading and listening comprehension

As introduced above, we conceptualize informal learning by focusing on how students engage with content in English as a foreign language during their leisure time in the present study. Such content is increasingly available for students in a globalized world, making it important for researchers to understand how students can utilize these opportunities for their language development.

For this purpose, we define language activities as the frequency with which students engage with EFL content (either by reading or by listening to content, for example, by reading books and magazines, or by listening to radio or audio recordings such as podcasts and audio books). Importantly, we focus on the activities that students engage in on their own time and not for school or homework-related purposes (Werquin, 2010; Greene et al., 2021). Only few of the studies focusing on how informal English activities relate to achievement outcomes have taken more than one specific type of informal learning activity—and the corresponding language subskills—into account (i.e., reading or listening skills; see Supplementary Table 1 in Supplementary material F). However, more studies focusing on such fine-grained perspectives that compare different modalities are important to enhance our understanding of the mechanisms at play in informal learning contexts.

The acquisition of reading and listening comprehension skills has been studied in the context of language competency modeling for formal learning (Gough et al., 1996). A significant aspect of this research has been the debate on whether cognitive processes involved in language comprehension should be considered modality-independent or modality-specific (Rost and Schilling, 2006). The role of modality in language acquisition still remains inadequately explored (Gilabert et al., 2016), and is characterised by inconsistent findings in prior literature (Zhao et al., 2021). For example, some studies suggest that auditory and visual inputs are processed separately in the brain (Siegelman and Frost, 2015).

Within the literature, hypotheses for both directions have been formulated: According to Coumel et al. (2023), it is conceivable that language learners might find visual input more accessible than auditory input, leading to possible implications for informal language learning. For instance, learners first need to decode speech when presented with spoken input, which can be challenging (Gilabert et al., 2016; Kim and Godfroid, 2019). In contrast, written language input allows learners to read sentences repeatedly and at their own pace, potentially fostering deeper linguistic intake processing. Due to the transitory nature of auditory input, higher attention is required, which might make visual stimuli more conducive to syntactic processing and language learning (Wong, 2001). Nonetheless, empirical evidence on the superiority of one modality over the other remains inconclusive, with some studies supporting auditory modality (Frost et al., 2019; Zhao et al., 2021; Coumel et al., 2023). As summarized by Coumel et al. (2023), other studies show no significant differences in learning across different input modalities (auditory/visual inputs). For instance, Morgan-Short et al. (2018) found no impact of input modality on Spanish as foreign language comprehension. Kim and Godfroid (2019) found that written and spoken language input resulted in similar learning outcomes regarding language, but written input was more effective for content knowledge. These variations in knowledge acquisition depending on modality might play a vital role in real-life settings, especially in informal language learning, where learners have more autonomy in choosing their activities. Hence, considering language modality becomes crucial when exploring the effects of informal language learning.

### 1.3 Self-concept, intrinsic value, and (informal) language learning

For the purpose of the present study, we define student motivation as “the process whereby goal-directed academic activity is instigated and sustained” (Koenka, 2020, p. 3; see also Schunk et al., 2014). More specifically, to conceptualize motivation, we use (situated) expectancy value theory (Eccles and Wigfield, 2002, 2020) as a widely established theoretical framework focused on motivation in academic domains. According to the SEVT (Eccles and Wigfield, 2002, 2020), expectancy beliefs, which closely relate to students’ self-concepts in domain-specific abilities and value beliefs predict the frequency of a certain type of behavior. In other words, the SEVT assumes that if students believe they can succeed at a given activity and become interested in it, they are more likely to engage in such a task (Eccles and Wigfield, 2002, 2020).

Based on these assumptions, SEVT defines (see Eccles and Wigfield, 2002, 2020) task values as subjective reasons for engaging in a certain task, which means that the same task can be valued differently by different students. These individual differences in academic motivation have been shown to be reflected in academic achievement across different academic domains (e.g., Jansen et al., 2015, 2016; Meyer et al., 2019; Fong et al., 2021). According to SEVT, the overall value of a task consists of different aspects, including intrinsic value (see Wigfield et al., 2017; Wigfield and Eccles, 2020). Intrinsic value is defined by Wigfield and Eccles (2000) as the anticipated enjoyment a student expects to experience when engaging in a task, and as the enjoyment a student experiences when actually doing so (Eccles and Wigfield, 2020). Even though motivational reactions can differ between situations, students’ self-concepts and value beliefs can describe tendencies toward a school subject or domain (e.g., Wan et al., 2021).

Based on SEVT (e.g., Eccles and Wigfield, 2020), it can be argued that engaging with English language content in informal ways can increase motivation in English as a foreign language. For example, making use of the English language in an informal way (such as reading an article or listening to a radio-show on a topic of interest) allows students to gain information not available in their native language, and thus gain more knowledge about the world, about ideas or events that they care about. Informal learning often occurs in contexts that are personally meaningful and interesting to the learner, which closely aligns with how SEVT conceptualizes aspects of task value (e.g., Eccles and Wigfield, 2020). When learners are able to relate English learning to their interests or real-life purposes, they might find more value in acquiring the language. This principle is often used in motivation interventions to foster students’ value beliefs of a specific task or domain (see Lazowski and Hulleman, 2016, for a review). Accordingly, informal language use is an activity that can elicit positive experiences during the activity, thus increasing intrinsic value of English learning. Further, engaging with English content in these real world situations can lead to greater feelings of competence, thus providing performance information and potentially increasing English self-concept (Davis-Kean et al., 2008; Weidinger et al., 2018). Thus, engaging in these tasks helps increase student English motivation as students can consider learning English as more fun while at the same time becoming more confident about their abilities to understand English content, which can also transfer to classroom tasks.

In summary, based on the conceptualizations of academic motivation from SEVT (e.g., Eccles and Wigfield, 2020), students’ English motivation can be influenced by informal learning experiences, that is, successful informal learning experiences may enhance both self-concept and intrinsic value. In a study addressing motivational consequences of informal language learning and focused on foreign language enjoyment, Lee and Lee (2021) found that Korean English learners benefited from informal language activities that resulted in greater enjoyment of language learning. To the best of our knowledge, prior studies on how informal language learning relates to self-concept and intrinsic value has focused mainly on smaller samples and college students. Less is known about the role of listening and reading activities from longitudinal studies.

### 1.4 The present study

**FIGURE 1:**
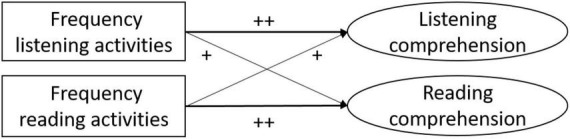
Graphical illustration of research question 1a. Indicates the size of the expected association with larger hypothesized effects depicted as ++.

This study investigated the extent to which upper secondary school students can benefit from engaging with English language content in informal ways during their leisure time. Our goal was to provide further evidence on the role of students’ informal reading and listening activities with English language achievement outcomes, substantiating evidence from previous research by using longitudinal data and a set of strong covariates to more precisely estimate the effects. We went beyond existing research in focusing specifically on effects of modality, leading to two main research questions. In Research Question 1 (RQ1), we focused on language achievement: we investigated how the frequency of students engaging in listening and reading activities as informal learning opportunities relates to English achievement. Looking at these associations in more detail, we investigated the role that modality plays in informal language learning (RQ1a), focusing on reading and listening. We assumed beneficial effects if the learning situation and measured skill had the same modality. Thus, we expected that a higher frequency in listening activities would relate to an increase in students’ development of listening comprehension and that a higher frequency in reading activities would relate to an increase in reading comprehension. For an illustration of the hypotheses for RQ1a, see Figure 1. In Research Question 1b, we focused on school grades in English as a foreign language, considering the role informal reading and listening activities might play for students’ end-of-the-year school grades, thus, using a more general and school-based measure of English achievement. Our hypothesis was that increased informal English language use would lead to higher school grades.

In Research Question 2 (RQ2), we focused on how the frequency of engaging with English language activities relates to students’ English self-concept and intrinsic value as important aspects of motivation according to SEVT (Eccles and Wigfield, 2020). We assumed that spending more time on listening and reading activities in English would relate to a more positive English self-concept and higher intrinsic value of English language learning. We investigated both hypotheses while controlling for a number of covariates, including prior achievement, prior motivation and prior frequencies of activities to estimate how informal learning activities relate to learning and motivational development in upper secondary education.

## 2 Materials and methods

### 2.1 Sample

We performed secondary data analyses with a data set made available upon request by the Research Data Centre of the Statistical Offices of the federal states (FDZ) in Berlin. We provide the data and analytic code to replicate our analyses at https://osf.io/2vkyc/. This study’s design and its analysis were not pre-registered. This study was reviewed by the Ministry of Education, Science and Cultural Affairs in Schleswig-Holstein. At the time of data collection, the ministry took sole responsibility for reviewing research ethics and privacy issues in all statewide research studies that took place in schools. The data set was originally generated in the context of the LISA study (Reading in Secondary Education; Measurements 5 and 6; see Leucht et al., 2016). This data set includes a representative sample of academic-track students in upper secondary schools from 82 classrooms from 21 schools. Measures were collected at two measurement time points, one in Grade 11 and on in Grade 13; in one German federal state (Schleswig-Holstein). The study took place in the years 2011 and 2013. The number of registered students was 1,880 at T1 and 1,433 at T2. All students who were registered for the study for at least one measurement point were included in the analysis. This resulted in a final longitudinal sample of 1,994 academic-track students (mean age at T1 = 16.8 years; 51.3% female; 93 percent native language German; 98 percent of students reported that they were born in Germany).

### 2.2 Study design

**FIGURE 2:**
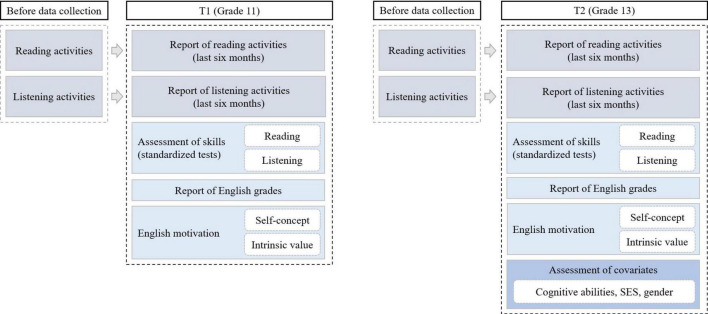
Overview of variables measured in the study.

We measured all relevant variables in Grade 11 and Grade 13 to allow to control for prior achievement, prior activities, and prior motivation. Figure 2 illustrates the study design, showing the measurement times of the variables.

### 2.3 Instruments

#### 2.3.1 English language activities

Activities of students engaging with English content were assessed with a self-report questionnaire at T1 and T2, asking about activities during leisure time within the last 6 months, adapted from Sachse et al. (2012). The items related to leisure time were focused specifically on students’ activities that were unrelated to their school activities or homework. We included items addressing reading [i.e., books, newspapers and magazines (one item)] and listening [i.e., audio books, radio (two items)]. We asked how often students had engaged in the activity in the last 6 months. A five-point response format was used, ranging from *never* to *more than five times*. We calculated a mean for activities related to listening. The original German items and their English translations can be found in Supplementary material A.

#### 2.3.2 English achievement: grade, reading, and listening comprehension

English achievement was assessed with tests that captured receptive language skills (i.e., reading and listening comprehension). Listening and reading comprehension tests were administered at both T1 and T2, using a subset of items from the German National Assessment (e.g., Stanat et al., 2016). The test items were designed to monitor the implementation of educational standards in Germany (see Köller et al., 2010) and therefore test skills based on the curricula for the English-language classroom. Three to four tasks consisting of multiple items (i.e., with the number of items per task ranging between three and nine) were presented in four 15-min blocks. Blocks were balanced in difficulty and rotated in eight different booklets to control for position effects and performance decline with test duration (multimatrix design). At T2, a subset of the same tasks was used. English grade was collected from school administration at T1 (end of year 10 grade) and T2 (end of year 13 grade).

As we conducted secondary data analyses, we used the scores from previous scaling procedures estimating English achievement in each listening and reading comprehension for each time point (see Leucht et al., 2015). To allow for comparability of proficiency scores between the two measurements, the drawing of plausible values for T2 was done by linking item parameters to those of T1 (see von Davier et al., 2008). As an estimate of student achievement in listening and reading comprehension we used the expected *a posteriori* (EAP) values derived from the IRT scaling procedures as a point estimator of the distribution of plausible values. EAP-reliabilities were satisfactory, ranging from 0.81 to 0.91 (see also Leucht et al., 2015). The reliability and validity of the test have been shown in previous studies; results can be linked to similar standardized tests such as PISA (see Fleckenstein et al., 2016).

#### 3.3.3 English self-concept and intrinsic value

We measured English motivation with three items on self-concept (e.g., “I have always been good at English”). Internal consistency was good with a Cronbach’s alpha of 0.82 at T1, and 0.88 at T2. We used two items to measure intrinsic value (e.g., “I look forward to English lessons”; see Eccles and Wigfield, 2002; Trautwein et al., 2012). For both self-concept and intrinsic value, we used a four-point Likert scale (*fully disagree* to *fully agree*). Internal consistency was satisfactory with a Cronbach’s alpha of 0.83 at T1, and 0.74 at T2. The original German items and their English translations can be found in Supplementary material A.

#### 3.3.4 Covariates

The following covariates were included in our study: SES, gender, cognitive ability, prior achievement in reading and listening (at T1), extramural activities in reading and listening (at T1), prior self-concept, prior intrinsic value, and prior English grade (all at T1). On the basis of our considerations, we investigated the associations of reading activities with the five outcome variables and the association of listening activities with the five outcome variables in separate models, because reading activities would be a colliding variable with listening activities if measured at the same time point, and vice versa.

Additionally, we considered several relatively time-stable covariates that were measured only at T2: cognitive ability, gender, and socioeconomic status (SES). General cognitive ability was measured using the figural and verbal reasoning subscales of the cognitive ability test (KFT4-12R; Heller and Perleth, 2000). The KFT4-12R is often used in the context of large-scale educational studies in Germany. EAP reliability was 0.80. Students’ SES was measured by parents’ occupational status in order to compute the Highest International Socio-Economic Index of Occupational Status (HISEI; Ganzeboom et al., 1992). Gender was assessed dichotomously (male and female) and collected from school administration lists. This means that gender is included as reported by the classroom teachers, and not as self-reported by students.

### 2.4 Statistical analyses

We applied structural equation modeling using M*plus* (Version 8; Muthén and Muthén, 1998–2017). First, we analyzed separate models for (a) listening activities and (b) reading activities as predictors of reading comprehension, listening comprehension, and English grade, respectively, to address RQ1. Second, we analyzed (a) listening activities and (b) reading activities as predictors of English motivation, that is, self-concept and intrinsic value, to address RQ2.

**FIGURE 3:**
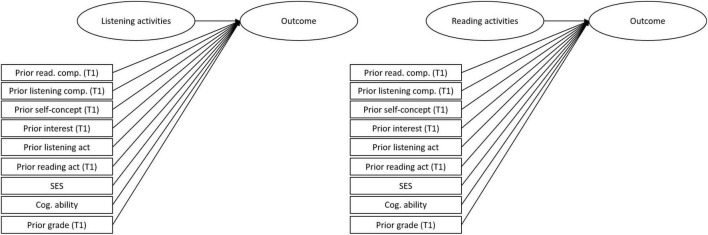
Illustration of statistical analyses. We computed separate models focusing on the predictive value of listening and reading. For simplicity, in the figure we only show one regression analysis for listening activities and one for reading activities (both assessed at T2). The term outcome refers to listening comprehension at T2, reading comprehension at T2 and English grade at T2.

Figure 3 illustrates our analytic design. As is visible, we estimated the association between reading and listening activities, respectively, and the development of reading and listening comprehension skills using a strong set of control variables. We used the R package *MplusAutomation* to prepare the data and run the models (Hallquist and Wiley, 2018). The models were based on maximum likelihood estimation with robust standard errors to account for dependencies in the data (i.e., students are nested in classrooms). All reported coefficients are fully standardized.

### 2.5 Missing data

As several students participated in only one of the measurements, the number of missing values on the questionnaire was high, with 32.6% (T1) and 64.5% (T2) for the items assessing the frequency of English language activities, and a range of 34% (T2) to 41% (T1) for the standardized tests. Supplementary material B provides analyses of the missing values, showing how students who did not participate in the questionnaire items on reading and listening activities during leisure time differed from students who participated. Missing values were handled using the Full Information Maximum Likelihood approach (FIML; see Enders, 2001).

## 3 Results

### 3.1 Bivariate correlations

We provide the bivariate correlations of all the variables in Supplementary material C (Supplementary Table 4). Descriptive statistics for all included variables can be found in Supplementary Table 3. Bivariate correlations showed moderate to high correlations between the different achievement measures, ranging from *r* = 0.38 between English grade at T1 and reading achievement at T1/T2 and *r* = 0.91 between reading and listening achievement at T2. We found small gender effects with more positive self-beliefs (*r* = 0.16/0.19 at T1/T2) and intrinsic value (*r* = 0.12/0.10) beliefs for girls. Boys reported more frequent listening activities in their leisure time compared to girls at both time points (*r* = −0.10 and *r* = −0.18). Cognitive abilities related negatively to the frequency of listening activities and positively to the frequency of reading activities with small effect sizes (*r* = −0.12 and *r* = 0.10, respectively).

### 3.2 How reading activities relate to achievement outcomes

In RQ1a we focused on listening and reading as specific subskills measured by standardized achievement tests, investigating the role of modality. Findings for reading activities indicate that the frequency of reading activities is positively related to both reading comprehension (β = 0.19, *SE* = 0.03, *p* < 0.001) and listening comprehension (β = 0.18, *SE* = 0.04, *p* < 0.001). This pattern of results did not replicate for listening activities. We found a small significant coefficient (β = 0.08, *SE* = 0.04, *p* = 0.041) showing positive associations for listening activities with listening comprehension. We found no significant coefficient for listening activities with reading comprehension (β = 0.06; *SE* = 0.04; *p* = 0.121).

Addressing RQ1b, we considered how informal learning with English language content related to students’ English grades. The pattern of results for standardized achievement replicated for grades, where we found a positive association of reading activities (β = 0.11; *SE* = 0.04, *p* = 0.005). Again, we found no support for an association of English grades with listening activities (β = 0.00; *SE* = 0.05, *p* = 0.995). Full results can be found in Table 1.

**TABLE 1:** Results research question 1 (full models).

	Listening activities									Reading activities								
	**Reading comp.**			**Listening comp.**			**English grade**			**Reading comp.**			**Listening comp.**			**English grade**		
	**Est.**	***SE***	***p***	**Est.**	***SE***	***p***	**Est.**	***SE***	***p***	**Est.**	***SE***	***p***	**Est.**	***SE***	***p***	**Est.**	***SE***	***p***
T2 Listening act.	0.06	0.04	0.121	0.08	0.04	0.041	0	0.05	0.995	/	/	/	/	/	/	/	/	/
T2 Reading act.	/	/	/	/	/	/	/	/	/	0.19	0.03	<0.001	0.18	0.04	<0.001	0.11	0.04	0.005
Prior read. comp. (T1)	0.23	0.04	<0.001	0.19	0.04	<0.001	0.15	0.03	<0.001	0.21	0.04	<0.001	0.17	0.04	<0.001	0.14	0.03	<0.001
Prior listening comp. (T1)	0.09	0.03	0.001	0.15	0.03	<0.001	0.07	0.03	0.039	0.09	0.03	0.001	0.15	0.03	<0.001	0.07	0.03	0.031
Prior self-concept (T1)	0.13	0.06	0.02	0.12	0.05	0.025	0.25	0.05	<0.001	0.13	0.06	0.018	0.12	0.05	0.022	0.24	0.05	<0.001
Prior interest (T1)	0.02	0.04	0.699	0.03	0.05	0.45	0.01	0.04	0.861	0.01	0.04	0.865	0.02	0.04	0.656	0	0.04	0.911
Prior listening act (T1).	−0.09	0.03	0.012	−0.06	0.03	0.095	−0.03	0.03	0.324	−0.09	0.03	0.003	−0.05	0.03	0.103	−0.04	0.03	0.091
Prior reading act. (T1)	0.08	0.03	0.003	0.06	0.03	0.036	0.07	0.03	0.033	0.03	0.03	0.311	0.02	0.03	0.639	0.04	0.04	0.262
Gender	0.03	0.03	0.24	0.02	0.03	0.367	0.01	0.03	0.689	0.04	0.03	0.164	0.03	0.03	0.324	0.02	0.03	0.439
SES	0.03	0.03	0.308	0.05	0.03	0.046	0.13	0.04	<0.001	0.02	0.03	0.489	0.05	0.03	0.074	0.12	0.04	0.001
Cog. ability	0.43	0.04	<0.001	0.38	0.03	<0.001	0.09	0.03	0.003	0.41	0.04	<0.001	0.37	0.03	<0.001	0.09	0.03	0.004
Prior grade (T1)	0.07	0.04	0.091	0.14	0.03	<0.001	0.25	0.05	<0.001	0.07	0.04	0.097	0.14	0.04	<0.001	0.26	0.04	<0.001
*R* ^2^	0.49	0.02	<0.001	0.52	0.03	<0.001	0.43	0.03	<0.001	0.52	0.02	<0.001	0.54	0.02	<0.001	0.44	0.03	<0.001

Read, reading; Act., activities; Cog. ability, cognitive ability; comp, comprehension; T1, Grade 11; T2, Grade 13; Gender 1, female, 0, male; SES, socioeconomic status.

Addressing RQ2, and the role of informal learning for motivational outcomes, we found evidence of a positive association of the reading activities with English self-concept (β = 0.24; *SE* = 0.04, *p* < 0.001) and intrinsic value (β = 0.17; *SE* = 0.05, *p* < 0.001). We also found positive associations of listening activities with English self-concept (β = 0.12; *SE* = 0.04, *p* = 0.004) and intrinsic value (β = 0.14; *SE* = 0.05, *p* = 0.003). Full results can be found in Table 2.

**TABLE 2:** Results research question 2 (full models).

	Listening activities						Reading activities					
	**Intrinsic value**			**Self-concept**			**Intrinsic value**			**Self-concept**		
	**Est.**	***SE***	***P***	**Est.**	***SE***	***p***	**Est.**	***SE***	***p***	**Est.**	***SE***	***p***
T2 Listening act.	0.14	0.05	0.003	0.12	0.04	0.004	/	/	/	/	/	/
T2 Reading act.	/	/	/	/	/	/	0.20	0.04	<0.001	0.25	0.04	<0.001
Prior reading comp. (T1)	−0.05	0.05	0.332	0.07	0.04	0.091	−0.06	0.05	0.226	0.06	0.04	0.198
Prior listening comp. (T1)	0	0.06	0.943	0	0.05	0.985	0	0.06	0.999	0	0.04	0.953
Prior self-concept (T1)	−0.04	0.1	0.649	0.58	0.07	<0.001	−0.04	0.09	0.684	0.57	0.07	<0.001
Prior interest (T1)	0.59	0.07	<0.001	0.06	0.05	0.241	0.57	0.07	<0.001	0.04	0.05	0.427
Prior listening act. (T1)	0.07	0.06	0.22	−0.05	0.04	0.221	0.10	0.05	0.058	−0.03	0.04	0.494
Prior reading act. (T1)	−0.10	0.05	0.055	0.01	0.04	0.84	−0.16	0.05	0.003	−0.05	0.04	0.143
Gender	0.03	0.05	0.519	0.03	0.04	0.468	0.02	−0.05	0.644	0.03	0.03	0.41
SES	0.06	0.05	0.252	0.04	0.04	0.258	0.06	0.05	0.266	0.04	0.03	0.258
Cognitive ability	−0.03	0.05	0.528	0.06	0.04	0.162	−0.06	0.05	0.207	0.04	0.04	0.41
Prior grade (T1)	0.14	0.07	0.031	0.1	0.05	0.049	0.15	0.07	0.02	0.11	0.05	0.021
*R* ^2^	0.41	0.05	<0.001	0.57	0.04	<0.001	0.43	0.05	<0.001	0.61	0.04	<0.001

Act., activities; Prior ach., prior achievement; Mot., motivation; T1, Grade 11; T2, Grade 13; Gender 1, female, 0, male; SES, socioeconomic status.

In summary, considering achievement outcomes as measured by standardized tests in the subskills of reading and listening as well as overall English grade, our findings supported a positive association of students’ informal activities with reading activities. By contrast the association of listening activities with these English competences was smaller. Concerning motivational outcomes, we found evidence of positive associations of both reading and listening activities with self-concept and intrinsic value.

## 4 Discussion

This study examined the relationship between informal English learning activities (reading and listening) on students’ English achievement and motivation. Given that the prior research was mainly either experimental, cross-sectional and within college samples, our study contributes to the evidence base by providing a longitudinal field study in German upper secondary school students. We found evidence that informal language activities predict the development of English achievement and motivation over time. By including a number of relevant covariates our results we offer a more precise estimate of the role of informal learning activities for formal learning contexts. The study has two central findings, addressing (a) the role of informal activities for English achievement and (b) English motivation, both of which we will discuss in the following.

### 4.1 Informal reading and listening activities and English achievement

First, our results provide evidence that upper secondary school students can benefit from informal reading of English language content during their leisure time. These associations held even after a set of strong potential confounders has been controlled for. Controlling for prior variables in addition to important covariates (gender, SES, and cognitive ability) allowed us to get an estimate of how much of the change in English achievement between Grade 11 and Grade 13 can be explained the frequency of engaging in reading activities. Our findings empirically consolidate the intuitive assumption that reading activities as informal learning opportunities provide promising means for students to increase their English achievement. This might be especially important in light of prior findings that learning gains in upper secondary school are usually smaller than those in lower secondary school (Hill et al., 2008; Keller et al., 2020). However, the effects were smaller for listening activities, where evidence was less consistent. We conclude that reading might be more beneficial for foreign language achievement outcomes compared to listening activities. One explanation could be that reading requires a higher level of attention and mental effort, especially in the context of informal learning. Students who read English literature books or magazines need to be fully focused to grasp the content, but students who are listening to audio books or radio might do so while performing other activities, such as walking or cooking, and thus might be less focused on the content. This could be an important insight in the context that teenagers tend to read fewer books while audio and video based internet sources are becoming ever more popular (Twenge et al., 2019).

In light of this alternative explanation and the positive association of listening activities with motivation we found in our study, we should not discard potentially beneficial effects of engaging in listening activities for language learning progress. To explain the differences in the case of grades, another explanation might be that listening skills are reflected less strongly in grades. As our findings do not reveal the mechanisms at play here, more research is needed to understand these patterns of results.

### 4.2 Informal reading and listening activities and English motivation

Turning to our second research question, our results suggest a different pattern for motivational compared to achievement outcomes. Here, we found that both reading and listening activities were positively related to students’ motivational development. For both modalities, we found positive associations for students’ English self-concept and intrinsic value. In other words, students who engaged more frequently with English language content, either by reading or by listening, became more motivated in learning English. This study thus substantiates evidence from previous research (Lee and Lee, 2021) that informally engaging with English media during leisure time can relate positively to students’ motivation development. We hope that our study encourages researchers to build on our findings in the future and consider in more detail how informal learning might be related to students’ motivation as an outcome of education in its own right. For example, studies focusing more on the situative aspects of English motivation might be interesting (see Pekrun and Marsh, 2022 for an overview of future perspectives on situated motivation): in the current study, we focused on more stable aspects of self-concept and intrinsic value measured in two different school years, but intensive longitudinal data collections and behavioral trace data could help better understand the interplay of informal language activities and students’ motivation.

All in all, our findings provide evidence that engaging with English language content in leisure time, especially by reading, might provide beneficial informal learning opportunities with positive associations in both performance and motivational outcomes. Note that our study had strong covariates, which strengthens the interpretability of our findings. However, we cannot speak of causal effects here as we only conducted an observational longitudinal study and did not manipulate any variables. Still, our study provides robust evidence of how informal learning benefits language achievement in an authentic classroom context over the course of two school years.

### 4.3 Implications for practice and future research

The results of this study are relevant for teachers and educational policies. It appears clear that informal reading in English is a powerful factor that can relate to the development of both achievement outcomes and motivation. Schools and educators can support informal reading, for example, by providing learners with suitable material (e.g., in situations in which learners do not have access to materials due to financial or social constraints). This could be achieved through free access to reading materials which is of interest to the students (magazines, young-adult novels, graphic novels, etc.). However, note that informal learning with English language content students’ engage with can supplement their English learning, but it should not be construed as a replacement of teaching in formal learning contexts.

Identifying alternative ways to enhance student achievement constitutes an important challenge for researchers, policymakers, and practitioners all around the globe. Our study adds to that knowledge and we hope that with our study, we could shed more light on how students can benefit from informal learning in language domains. However, more research is needed comparing the beneficial role of informal learning with other ways of effectively teaching language, such as immersion programs or other types of instruction, to understand which aspect of formal and informal learning opportunities might have the most impact on both students competencies as well as their motivation across different domains (e.g., Freed et al., 2004).

The exact mechanisms through which reading and listening activities have positive effects remain unclear in our findings and should be addressed in further research. From our perspective, multiple plausible explanations for our pattern of results exist. First, spending more time to engage with English language content might be beneficial due to increased “time on task” (Scheerens, 2014) and other measures of student engagement (Wong et al., 2023) which relates to increased academic achievement. This assumption is also reflected in the self-teaching hypothesis by Share (1995, 2008), who argued that by phonological recoding of language content students independently (i.e., by self-teaching) acquire new language skills (e.g., an autonomous orthographic lexicon), supporting reading acquisition. Research on self-teaching has mostly involved L1 learners, but there is also evidence of childrens’ self-teaching in the context of orthographic learning in an EFL context (Schwartz et al., 2014; for a recent review of the literature see also Li and Wang, 2023). Our study adds to that body of literature, but more research is needed to better understand the mechanisms of this effect. For example, benefits might be due to an enhanced learning motivation of students who engage with English language content in their free time. Another explanation is that students who engage in these activities have a more privileged home environment, allowing them greater access to these opportunities (see Gottfried et al., 1998; Arnold and Doctoroff, 2003), or more supportive parents encouraging them to engage in these activities. We aimed to control for these possible influence by using SES as a covariate, but more variance might be explained with more nuanced measures, for example reflecting parents’ academic attitudes.

## 5 Limitations

Some further issues associated with our study need to be acknowledged and addressed in future research. First, this study was based on observational field data, with our measures of students’ leisure time activities being questionnaire instruments and only including a small number of items, possible limiting the reliability of the results. The measures can be criticized as they quite broadly assess how often students engage with English language content by listening and reading in their leisure time. To increase the understanding of why positive associations were found in our study, more process-oriented questions on what happens during informal language learning with English language content should be addressed in future studies. This might include multimethod approaches, including focus groups asking students in detail how they use different media, and why and how they engage with English language content.

Relatedly, we assessed the students’ behavior regarding their engagement with informal learning activities with English language content retrospectively, that is, the instruments focused on the 6 months prior to the second data collection. Notably, we also controlled for learning activities at the first measurement time point (see Figure 3), which was argued to constitute a particularly helpful technique to rule out reverse causation (Vanderweele et al., 2020). However, doing so does not rule out the possibility of recall biases that might have affected the validity and reliability of our results. Also, such a retrospective questionnaire approach provides a rather broad measure, as behavior can change across 6 months and students might not fully recall their activities in the relevant time period. One way to address these potential shortcomings, which, however, would require great additional efforts, would be to implement an experience sampling design that assesses micro longitudinal data multiple times throughout the school year in order to gain more precise estimates of the extent to which students engaged in informal language activities. Future research could also capitalize on the potential of digital technologies for data collection and use behavioral indicators of students’ media behavior, for example, clickstream data from social media platforms to better understand how students’ informal leisure time activities might relate to their learning outcomes.

More specifically, we want to address how these issues with reliability and validity in the current data set might have biased our results: for example, it is possible that higher performing or more cognitively able students can recall their prior activities more precisely than lower performing or cognitively less able students, or that they can estimate the frequency of their previous activities more accurately. Thus, our results might reflect the association of informal learning and achievement more accurately for specific subgroups of students. We aimed to address these shortcomings by controlling for relevant covariates, including cognitive ability, and prior English achievement and, most importantly, prior activities.

On a related note, we only used three items for English self-concept, and two items for intrinsic value. The low number of items might somewhat reduce the validity of our findings regarding the motivational outcomes. However, it is quite common to use short scales in large-scale data collections for economic reasons. Second, our study was limited to the investigation of activity frequency. It is also plausible to assume that the duration or the intensity of these activities plays a role. A student who reads for 2 h at a time might experience stronger positive associations with achievement and motivation development than a student who engages in the activity as frequently, but only for 30 min at a time. Including the duration of engagement in the activity in an assessment could thus obtain information that would explain more variance between students. This could be an interesting question for future research, especially as students, parents, or educators could be informed about whether students would benefit more from engaging with English language content more often or for longer. Similarly, with an appropriate design, future studies could investigate whether the intense engagement with of English language content in different modalities can improve English achievement more than a long duration in the same modality. Results from such studies could be compared against our results, shedding new light on the processes of informal language learning.

Third, there were some more methodological limitations: we had a large number of missing values on the questionnaire items assessing the frequency of activities, especially at T2. This was due to the voluntary nature of the questionnaire, and our decision to include all students who were part of the study on at least one of the two time points to account for (potentially selective) dropouts. We compared students who participated in the questionnaire with students who did not for each of the time points. Results show that our sample was somewhat positively selected: students who participated in the questionnaire scored lower on English listening and reading tests, had higher self-concepts, and cognitive ability scores. Effect sizes were small. Full results can be found in Supplementary material B.

Fourth, for the present study we included only activities that could be clearly labeled as reading or listening activities. However, it should be mentioned that especially newer media channels (e.g., streaming films or gaming in an interactive online environment) provide learners with content in different modalities, often at the same time. When playing computer games, for example, users can encounter written input through instructions and dialog on the screen and spoken input through audio dialog. Interactive games might even require players to engage in spoken interactions with other players in audio (-visual) chats. Watching movies can expose learners to audio-visual as well as written input through subtitling, and modern music apps can play songs while simultaneously displaying the lyrics at the bottom of the screen. Unfortunately, an investigation of these various further modalities (or combinations of modalities) was not feasible for this study as the dataset did not contain information on specific activities that students engaged in when they were using these media. Accordingly, we do not know whether learners used subtitles and were thus unable to formulate specific hypotheses for watching movies as informal activity regarding the association with reading and listening skills. It also remains unclear whether and to what extent it is possible to clearly isolate individual modalities of modern media channels, which would be important in order to estimate their unique association with reading and listening comprehension.

On a related note, there are issues concerning the response format of the items measuring the extent to which students engaged with English language content by reading or listening relating to the rapidity of developments in accessibility of English language media in the past few years. Because of these changes in technology and media, data sets and even questionnaires quickly become outdated. We used a data set from 2011 to 2013. At that time, students already engaged with the internet and electronic media on a regular basis. Furthermore, online radio and English books were already available to a broader audience. However, since then, more and more content has become a part of students’ daily lives, especially in social media. Accordingly, more research is needed that focuses on younger populations in order to investigate whether our findings would still hold despite ongoing changes in the increasing availability of English media. For example, online streaming became more popular in Germany in 2014. Thus, we believe that the instruments we used with the respective response format were appropriate in the specific cohort at the time of the data collection, but future research would need to adapt the measures to reflect changes in the availability of English media. Additionally, future research should consider the impact of generative artificial intelligence on students’ language competencies (e.g., Kasneci et al., 2023), as these technologies provide new opportunities for informal learning and self-teaching, for example by generating input that can explain other materials. They could thus support lower-performing students in their self-teaching not only in the school-context, but also during informal learning.

In summary, our study adds to the body of research on English as a foreign language informal learning by utilizing a large-scale longitudinal data set and a strong set of covariates. Further, we provide a standardized assessment of English language competencies, considering both listening and reading comprehension using items from established standardized tests, and using multiple outcome measures. However, there were some issues with measurement of extramural learning, especially regarding the retrospective and self-reported nature of the informal learning activities.

## 6 Conclusion

This study investigated the extent to which engaging with English language content in informal contexts can be beneficial for upper secondary school students’ English as a foreign language achievement and motivational development. Using a large-scale longitudinal data set of German students, we found positive associations between students’ informal reading activities and English as a foreign language achievement, highlighting how informal opportunities to engage with English language content might benefit students’ English proficiency even in formal learning (and achievement) situations. For motivation, we found that engaging with the content by either reading or listening had positive associations with both self-concept and intrinsic value. As our study used longitudinal data and strong controls (i.e., prior measures of the outcome and treatment variables), the results provide a useful extension to prior research in this field.

## Data availability statement

We performed secondary data analyses with a data set made available upon request by the Research Data Centre of the Statistical Offices of the federal states (FDZ) in Berlin. We also provide the data and analytic code to replicate our analyses at https://osf.io/2vkyc/.

## Ethics statement

The studies involving humans were approved by the Ministry of Education, Science and Cultural Affairs in Schleswig-Holstein. The studies were conducted in accordance with the local legislation and institutional requirements. Written informed consent for participation in this study was provided by the participants’ legal guardians/next of kin.

## Author contributions

JM: Conceptualization, Formal analysis, Writing – original draft, Writing – review and editing. JF: Writing – review and editing. MK: Writing – review and editing. SK: Writing – review and editing. NH: Conceptualization, Formal analysis, Writing – original draft, Writing – review and editing.
