# Supplementary material for: Read at home to do well at school: informal reading predicts achievement and motivation in English as a foreign language
Source: Front Psychol. 2024 Jan 23;14:1289600. doi: 10.3389/fpsyg.2023.1289600 (PMC10844388; doi:10.3389/fpsyg.2023.1289600)
Supplement: Supplementary file 1 [file Table_1.pdf]

Supplemental Material

Supplement A

Original items and English translation for each questionnaire instrument

	German	English translation by the first author
Self-Concept English item 1	Englisch ist eines meiner besten Fächer	English is one of my best subjects.
Self-Concept English item 2	Ich war schon immer gut in Englisch	I have always been good at English.
Self-Concept English item 3	Im Fach Englisch bekomme ich gute Noten	In English I get good grades.
Intrinsic Value English item 1	Ich hätte gern mehr Englisch Stunden	I would like to have more English lessons
Intrinsic Value English item 2	Ich freue mich immer auf die Englisch-Stunden	I am always looking forward to my English lessons.
Reading activities	Wie oft haben Sie im letzten halben Jahr Folgendes in Ihrer Freizeit getan? Ich habe Bücher, Zeitungen oder Zeitschriften in englischer Sprache gelesen	How often did you engage in the following activities during leisure time within the last 6 months? I read books, newspapers or magazines in English language.
Listening activities item 1	Wie oft haben Sie im letzten halben Jahr Folgendes in Ihrer Freizeit getan? Ich habe Radiosendungen in englischer Sprache gehört	How often did you engage in the following activities during leisure time within the last 6 months? I listened to radio shows in English language.
Listening activities item 2	Wie oft haben Sie im letzten halben Jahr Folgendes in Ihrer Freizeit getan? Ich habe Hörbücher auf Englisch gehört.	How often did you engage in the following activities during leisure time within the last 6 months? I listened to audio books in English language.

Supplement B: Selectivity Analyses

Table S2

Logistic Regressions Including Variables Used in the Analyses Predicting Whether Students did not Participate in the Listening and Reading Activities Questionnaire at T1 or T2, Respectively

Missing T1	Estimate	SE	p-value	Missing T2	Estimate	SE	p-value
Intrinsic value T2	.00	.04	.948	Intrinsic value T2	-.04	.10	.691
Intrinsic value T1	.09	.05	.077	Intrinsic value T1	-.01	.03	.774
Self-concept T1	.05	.05	.247	Self-concept T1	.07	.03	.012
Self-concept T2	-.03	.04	.458	Self-concept T2	.01	.07	.896
Listening comprehension T2	.01	.03	.616	Listening comprehension T2	.12	.03	<.001
Listening comprehension T1	.03	.04	.357	Listening comprehension T1	-.01	.04	.820
Reading comprehension T1	.08	.03	.013	Reading comprehension T1	.01	.03	.679
Reading comprehension T2	.01	.03	.741	Reading comprehension T2	.11	.03	.001
SES	-.04	.03	.194	SES	-.05	.04	.241
Cognitive ability	.03	.03	.277	Cognitive ability	.07	.03	.027
Gender	.17	.08	.038	Gender	.03	.04	.341

Note. Students who had missing values on more than half of the listening and reading activities in the questionnaire at T1 or T2, respectively were coded with 0, and students who participated were coded with 1. Variables were used to predict whether students had missing values in a logistic regression. We report standardized coefficients. Gender female =1; male = 0.

Supplement C: Robustness Checks

Table S3

Results Full Models Using Reading Activities T2 As Predictor

	Reading comp.			Listening comp.			Intrinsic value			Self-concept			English grade		
	Est.	*SE*	*p*-value	Est.	*SE*	*p*-value	Est.	*SE*	*p*-value	Est.	*SE*	*p*-value	Est.	*SE*	*p*-value
**Reading act. T2**	.17	.03	<.001	.15	.03	<.001	.17	.05	<.001	.24	.04	<.001	.09	.04	.022
Prior reading comp.	.25	.04	<.001	.19	.04	<.001	-.06	.05	.245	.06	.04	.193	.12	.04	<.001
Prior listening comp.	.09	.03	.003	.15	.04	<.001	.00	.06	.974	-.01	.04	.896	.07	.04	.057
Prior self-concept	.17	.03	<.001	.12	.06	.04	-.06	.10	.567	.56	.08	<.001	.23	.06	<.001
Prior interest	.02	.05	.646	.03	.05	.473	.57	.07	<.001	.06	.06	.275	-.02	.05	.732
Prior listening act.	-.07	.03	.021	-.01	.03	.714	.10	.06	.066	-.04	.04	.341	-.01	.03	.680
Prior reading act.	.00	.03	.956	-.02	.04	.625	-.15	.06	.011	-.04	.04	.230	.04	.04	.282
Gender	.03	.03	.323	.02	.03	.598	.03	.05	.537	.01	.04	.711	.02	.03	.601
SES	.01	.03	.818	.04	.03	.172	.06	.05	.247	.04	.03	.223	.14	.04	<.001
Cog. Ability	.45	.04	<.001	.41	.04	<.001	-.03	.05	.546	.06	.05	.225	.07	.04	.065
Prior grade English	.04	.04	.328	.14	.04	<.001	.15	.07	.024	.11	.05	.024	.29	.05	<.001
R^2	.57	.02	<.001	.59	.03	<.001	.39	.05	<.001	.603	.042	<.001	.45	.03	<.001

Note. Cog. = cognitive; Comp. = comprehension; Act. = activities; Prior ach. = prior achievement; Mot. = motivation; T1 = Grade 11; T2 = Grade 13; Gender 1 = female, 0 = male; SES = socioeconomic status;

Table S4

Results Full Models Using Listening Activities T2 As Predictor

	Reading comp.			Listening comp.			Intrinsic value			Self-concept			English grade		
	Est.	*SE*	*p*-value	Est.	*SE*	*p*-value	Est.	*SE*	*p*-value	Est.	*SE*	*p*-value	Est.	*SE*	*p*-value
**Listening act. T2**	.06	.04	.142	.07	.04	.074	.09	.04	.039	.11	.05	.026	-.03	.05	.591
Prior reading comp.	.26	.04	<.001	.20	.04	<.001	-.04	.05	.359	.08	.04	.085	.15	.04	<.001
Prior listening comp.	.09	.03	.004	.15	.04	<.001	.01	.06	.932	.00	.05	.969	.07	.04	.073
Prior self-concept	.15	.06	.02	.12	.06	.037	-.06	.11	.572	.56	.08	<.001	.23	.06	<.001
Prior interest	.03	.05	.492	.05	.05	.310	.58	.07	<.001	.08	.06	.160	-.01	.05	.773
Prior listening act.	-.07	.04	.050	-.02	.03	.652	.09	.06	.13	-.06	.04	.194	.01	.04	.856
Prior reading act.	.04	.03	.178	.02	.03	.550	-.10	.06	.086	.02	.04	.635	.07	.04	.073
Gender	.02	.03	.400	.01	.03	.621	.03	.05	.496	.01	.04	.761	.00	.03	.891
SES	.01	.03	.637	.05	.03	.128	.06	.05	.236	.04	.04	.230	.15	.04	<.001
Cog. Ability	.45	.05	<.001	.42	.04	<.001	-.01	.06	.858	.09	.05	.090	.07	.04	.058
Prior grade English	.04	.04	.336	.14	.03	<.001	.15	.07	.035	.10	.05	.053	.29	.05	<.001
R^2	.55	.02	<.001	.58	.03	<.001	.38	.04	<.001	.57	.04	<.001	.44	.03	<.001

Note. Cog. = cognitive; Comp. = comprehension; Act. = activities; Mot. = motivation; T1 = Grade 11; T2 = Grade 13; Gender 1 = female, 0 = male; SES = socioeconomic status;

Table S5

Bivariate Correlations (Robustness checks)

	Eng Grade T2	Eng. Grade T1	Gender	SES	Self-concept T1	Self-concept T2	Cogn. ability	Read. ach. T1	Read. ach. T2	List. ach. T1	List. ach. T2	Int. value T1	Int. value T2	List. act. T1	Read. act. T1	List. act. T2
English grade	.57***															
Gender	.05**	.22***														
SES	.21***	.08*	-.03													
Self-concept T1	.49***	.66***	.18***	.04												
Self-concept T2	.68***	.57***	.13**	.10*	.72***											
Cognitive ability	.20***	.15***	-.09*	.05	.02	.12**										
Reading achiev. T1	.44***	.39***	.02	.09*	.39***	.38***	.32***									
Reading achiev. T2	.51***	.38***	.03	.08*	.37***	.51***	.58***	.56***								
Listening achiev. T1	.43***	.43***	.07	.08*	.43***	.38***	.28***	.60***	.47***							
Listening achiev. T2	.58***	.46***	.04	.12***	.42***	.59***	.55***	.55***	.90***	.53***						
Intrinsic value T1	.26***	.39***	.10*	.07	.68***	.52***	-.04	.21***	.23***	.26***	.27***					
Intrinsic value T2	.32***	.32***	.10	.11*	.40***	.57***	-.03	.11**	.24***	.17***	.30***	.59***				
Listening act. T1	.12**	.07	-.12***	.07*	.19***	.14***	-.04	.06	.02	.12**	.08	.25***	.21***			
Reading act. T1	.28***	.22***	-.01	.13***	.36***	.30***	.01	.17***	.18***	.22***	.21***	.39***	.19***	.48***		
Listening act. T2	.06	.04	.10*	.10*	.14***	.18***	-.12*	.02	.01	.01	.05	.13*	.16***	.44***	.29***	
Reading act. T2	.25***	.13**	-.09*	.10*	.23***	.38***	.12**	.17***	.24***	.15***	.25***	.24***	.26***	.31***	.42***	.45***

Note. Eng. = English; List. = listening; read. = reading; int. value = intrinsic value; achiev./ach. = achievement; act. = activities; cogn. ability = cognitive ability; SES = socioeconomic status; SC = self-concept; Gender was coded with 1 = girls, 0 = boys.

Table S6

Additional Analyses: Predicting Reading and Listening Activities T2

	Reading activities T2			Listening activities T2		
	Est.	*SE*	*P*	Est.	*SE*	*p*
Prior reading comp.	.09	.06	.111	.03	.06	.652
Prior listening comp.	-.01	.05	.780	-.07	.05	.193
Prior self-concept	.06	.09	.521	.15	.09	.078
Prior intrinsic value	.04	.07	.608	-.10	.08	.200
Prior listening activities	.10	.04	.019	.36	.05	<.001
Prior reading activities	.33	.05	<.000	.10	.05	.048
Gender	-.09	.04	.017	-.17	.04	<.001
SES	.04	.05	.378	.06	.04	.131
Cog. Ability	.05	.04	.181	-.08	.05	.063
Prior grade	.03	.06	.691	.01	.06	.849
R^2	.24			.24		

Note. Cog. = cognitive; comp. = comprehension

Supplement D: Bivariate Correlations

Table S8

Bivariate Correlations

	Eng Grade T2	Eng. Grade T1	Gender	SES	Self-concept T1	Self-concept T2	Cogn. ability	Read. ach. T1	Read. ach. T2	List. ach. T1	List. ach. T2	Int. value T1	Int. value T2	List. act. T1	Read. act. T1	List. act. T2
English grade	.55***															
Gender	.12***	.23***														
SES	.21***	.11***	-.03													
Self-concept T1	.54***	.66***	.19***	.08*												
Self-concept T2	.72***	.56***	.16***	.13***	.72***											
Cognitive ability	.21***	.14***	-.07*	.04	.02	.09*										
Reading achiev. T1	.44***	.38***	.06	.07*	.40***	.36***	.31***									
Reading achiev T2	.54***	.38***	.06	.10**	.37***	.50***	.53***	.52***								
Listening achiev T1	.42***	.40***	.08*	.08*	.42***	.35***	.26***	.59***	.44***							
Listening achiev T2	.61***	.45***	.07*	.14***	.42***	.56***	.50***	.52***	.91***	.50***						
Intrinsic value T1	.34***	.38***	.12***	.09*	.67***	.50***	-.02	.24***	.22***	.27***	.27***					
Intrinsic value T2	.42***	.32***	.10*	.13**	.42***	.58***	-.05	.11**	.23***	.16***	.29***	.60***				
Listening activities T1	.10**	.10*	-.10***	.09**	.21***	.15***	-.06	.04	.02	.01	.06	.25***	.21***			
Reading activities T1	.27**	.15***	.02	.16***	.35***	.30***	-.02	.17***	.19***	.22***	.22***	.38***	.20***	.48***		
Listening activities T2	.08	.06	-.18***	.12**	.15***	.20***	-.12**	.01	.03	.01	.06	.11*	.20***	.43***	.29***	
Reading activities T2	.27***	.23***	-.07	.13***	.24***	.39***	.10**	.16***	.27***	.15***	.28***	.23***	.29***	.29***	.44***	.46***

Note. Eng. = English; List. = listening; read. = reading; int. value = intrinsic value; achiev./ach. = achievement; act. = activities; Cogn. Ability = cognitive ability; SES = socioeconomic status; SC = self-concept; Gender was coded with 1 = girls, 0 = boys.

Table S9

Descriptives

	Mean	SD	N	Min-Max
English grade T2	9.30	2.80	1413	1-15
English grade T1	2.53	0.81	876	1-5
Gender	0.51	0.50	1879	0-1
SES	62.41	7.90	808	14.21-88.96
Cognitive ability	0.33	0.57	1433	-2.07-1.90
Reading achievement T1	635.42	65.51	1171	398.40-815.56
Reading achievement T2	656.77	58.14	1317	459.54-809.83
Listening achievement T1	646.45	57.70	1171	427.72-811.50
Listening achievement T2	726.74	54.74	1317	525.83-863.19
Intrinsic value T1	2.51	.87	1417	1-4
Intrinsic value T2	2.18	.81	722	1-4
Self-concept T1	2.75	.78	1419	1-4
Self-concept T2	2.76	.86	719	1-4
Listening activities T1	1.73	36.32	1.731	1-5
Reading activities T1	2.877	1.69	1337	1-5
Listening activities T2	1.74	26.36	1.742	1-5
Reading activities T2	3.26	1.80	707	1-5

Note. Descriptives for intrinsic value and self-concept were computed as manifest scores.

Supplement E: Illustrative Overview of the Empirical Literature

Table S1

Illustrative Overview of the Empirical Literature Providing Evidence for the Role of Informal EFL Learning for Language Proficiency, Including Study Characteristics

Study	Study design	N	Age group	Country of study	Activity type	Skill
Fievez et al., 2023	Experimental	102	University	Dutch	TV	Vocabulary
Frumuselu et al., 2015	Experimental	40	University undergraduates	Spain	Subtitles	Vocabulary learning and film comprehension
González-Fernández & Schmitt, 2015	Observational	108	Adults	Spain	Reading	Vocabulary/ collocations
Jensen, 2017	Observational	107	Primary school	Denmark	Gaming, music, TV	Vocabulary
Kuppens, 2010	Observational	374	Primary school	Flemish	TV; online games	Oral translation
Kusyk & Sockett, 2012	Observational	45	University	French	TV	Oral comprehension of phrases (vocabulary)
Lai et al., 2015	Observational	82	Middle school	China	Out of class learning	English grades
Lee & Drajati, 2019	Observational	183	University	Indonesia	Digital activities	Motivation
Lee & Dressman, 2018	Observational	94	University	Korea	Digital activities	Motivation, speaking, vocabulary
Lee, 2019	Observational	77	University	Korea	Digital activities	Vocabulary
Lindgren & Munoz, 2013	Observational	865	Elementary school students	Multi country (Croatia, England, Italy, the Netherlands,	TV	Listening and reading comprehension

				Poland, Spain, Sweden)		
Niitemaa, 2020	Observational	46	Upper secondary	Finnish	Multiple online activities	Vocabulary recognition
Peters & Webb, 2018	Experimental	63	First or second year university students	Dutch	TV	Vocabulary
Peters, 2018	Observational	79	Secondary/university	Flemish	TV	Vocabulary
Puimège & Peters, 2019	Experimental	20	University	Dutch	TV	Vocabulary
Pujadas & Munoz, 2019	Experimental	106	Lower secondary	Spain	TV	Vocabulary
Rodgers, 2013	Experimental	321	University students	Japan	TV	Vocabulary, comprehension (meaning)
Schmitt & Redwood, 2011	Observational	68	mixed	England	reading	Vocabulary (phrasal verbs)
Sundqvist & Wikström, 2015	Observational	80	Lower secondary	Swedish	Gaming	Vocabulary, oral proficiency
Sylvén & Sundqvist, 2012	Observational	86	Elementary school	Swedish	Gaming	Vocabulary
Verspoor et al., 2011	Observational	240/ 316	Lower secondary school	Dutch	Out of school contact	Reading, vocabulary, writing
